# Pleural Empyema Due to Proteus Mirabilis in an Adult: A Rarely Encountered Clinical Scenario

**DOI:** 10.7759/cureus.36690

**Published:** 2023-03-26

**Authors:** Srujana Mohanty, Prasanta R Mohapatra, Debopam Chatterjee, Palanisamy Venkatachalam

**Affiliations:** 1 Microbiology, All India Institute of Medical Sciences, Bhubaneswar, Bhubaneswar, IND; 2 Pulmonary Medicine and Critical Care, All India Institute of Medical Sciences, Bhubaneswar, Bhubaneswar, IND

**Keywords:** gram-negative rod, malignancy, thoracocentesis, proteus mirabilis, empyema

## Abstract

The presence of *Proteus *species in the pleural space is an uncommonly reported entity and is rarely seen even in patients with compromised immune status. We report a case of pleural empyema due to *Proteus *species in an adult oral cancer patient receiving chemotherapy for academic interest and for generating awareness regarding an expanded pathogenic spectrum of the organism. A 44-year-old salesman, non-smoker and non-alcoholic, presented with sudden-onset shortness of breath, left-sided chest pain, and low-grade fever of one-day duration. He had been recently diagnosed with adenocarcinoma of the tongue and had received two cycles of chemotherapy. After clinical and radiographic evaluation, the patient was diagnosed with left-sided empyema. Following thoracocentesis, the aspirated pus sent for bacterial culture yielded pure growth of *Proteus mirabilis*. Appropriately modified antibiotic therapy with parenteral piperacillin-tazobactam followed by cefixime, tube drainage, and other supportive therapy resulted in a favorable outcome. After three weeks of hospitalization, the patient was discharged for further planned management of his underlying condition. Though uncommon, the possibility of *Proteus* species should be kept in mind as a causative agent of thoracic empyema in adults, especially in immunocompromised patients with cancer, diabetes, and renal diseases. The so-called common microorganisms of empyema appear to have altered over time, influenced by anticancer therapy and underlying host immune status. Rapid diagnosis and appropriate antimicrobial therapy usually result in a favorable outcome.

## Introduction

*Proteus* species are non-lactose fermenting, facultatively anaerobic, gram-negative motile rods that belong to the order Enterobacterales and commonly exist as normal inhabitants of the gastrointestinal tract [1]. These are easily identified by their classic “swarming” appearance on culture media (associated with the conversion of short swimmer cells into highly elongated hyper-flagellated swarmer cells) and distinct “chocolate cake” or “burnt chocolate” smell; the genus has four named species known to cause human clinical infections, such as *Proteus mirabilis*, *Proteus vulgaris*, *Proteus​​​​​​​ penneri*, and *Proteus​​​​​​​ hauseri* [1,2]. *P. mirabilis* is the most frequently encountered species responsible for 80%-90% of all *Proteus* infections in man [2].

Despite possessing various virulence factors, the *Proteus *species bacteria are mostly considered as opportunistic human pathogens, with infections observed mainly in people with an impaired immune system, such as those with structurally abnormal urinary tracts, previous use of antibiotics, corticosteroids or antineoplastic therapy, type 2 diabetes mellitus, carcinoma of colon, and those who are undergoing long-term catheterization [2,3]. The most common site of infection is the urinary tract; however, it has also been isolated from wounds and ears, and only occasionally from patients with diarrhea, sepsis, and endocarditis [2,3]. The presence of *Proteus* species in the pleural space is an uncommonly reported entity and is rarely seen, even in patients with compromised immune status. We report a case of pleural empyema due to *P. mirabilis* in an adult patient with underlying malignancy for academic interest and for generating awareness regarding an expanded pathogenic spectrum of the organism.

## Case presentation

A 44-year-old salesman, non-smoker and non-alcoholic, was admitted to the emergency department with complaints of sudden-onset shortness of breath, left-sided pleuritic chest pain, and low-grade fever of one-day duration. There was no history of trauma or injury to the chest. He was not diabetic or hypertensive and had no history of contact with tuberculosis. However, he had been recently diagnosed with adenocarcinoma tongue (Stage IVA) and had received two cycles of chemotherapy with paclitaxel, cisplatin, and 5-fluorouracil. Clinical examination revealed a thin-built man, conscious and oriented, with pallor and lymphadenopathy with a hard, round, fixed, left level 1B/2B node size. Examination of the oral cavity showed trismus grade 1 and a large ulceroproliferative lesion on the tongue extending from the tip anteriorly and involving the entire tongue, including the base of the tongue and floor of the mouth, suggestive of a locally advanced carcinoma of left lateral border of the tongue with fixed left level 2B lymphadenopathy. The patient had a blood pressure of 106/70 mmHg, pulse rate of 100/min, respiratory rate of 22/min, temperature of 39.2°C, and SpO_2_ of 94%. Chest examination showed diminished vesicular breath sounds with dullness to percussion over the left lower chest (infrascapular area) and moderate tenderness of the left upper quadrant.

Laboratory evaluation showed an extremely high total leukocyte count with marked neutrophilia (96%), low hematocrit, low erythrocyte count, and low hemoglobin level (Table 1). A peripheral blood smear was suggestive of severe anemia with predominantly normocytic normochromic red cells with few microcytic red cells. Other laboratory parameters were deranged including raised alkaline phosphatase, raised serum urea level, raised creatinine, low sodium, low chloride, and low albumin (Table 1). The patient tested seronegative for anti-HIV-1/2 antibodies, anti-hepatitis C virus (anti-HCV) antibodies, and hepatitis B surface antigen.

**Table 1:** Summary of laboratory investigations on admission

Laboratory test	Result	Reference range
Hemoglobin	84 g/L	130-170 g/L
Total red blood cell count	3.0 × 10^12^/L	4.5-5.5 × 10^12^/L
Hematocrit (packed cell volume)	25.4%	39.0%-51.0%
Mean cell volume	84.7 fL	81-101 fL
Mean cell hemoglobin	28.0 picogram	27-32 picogram
Mean cell hemoglobin concentration	331 g/L	310-340 g/L
Total leukocyte count	55.4 × 10^9^/L	4-11 × 10^9^/L
Serum urea	96 mg/dL	17-43 mg/dL
Serum creatinine	2.4 mg/dL	0.7-1.3 mg/dL
Serum uric acid	7.1 mg/dL	3.6-7.7 mg/dL
Serum sodium	121 mEq/L	135-145 mEq/L
Serum potassium	4.74 mEq/L	3.5-5.0 mEq/L
Serum chloride	88 mEq/L	95-110 mEq/L
Serum bilirubin (total)	0.8 mg/dL	0.3-1.2 mg/dL
Serum aspartate aminotransferase	18 U/L	5-50 U/L
Serum alanine aminotransferase	15 U/L	5-50 U/L
Serum alkaline phosphatase	212 U/L	30-120 U/L
Serum total proteins	63 g/L	67-86 g/L
Serum albumin	29 g/L	35-52 g/L
Serum globulin	34 g/L	27-45 g/L
Albumin: globulin ratio	0.9	0.8-2.0

**Figure 1:**
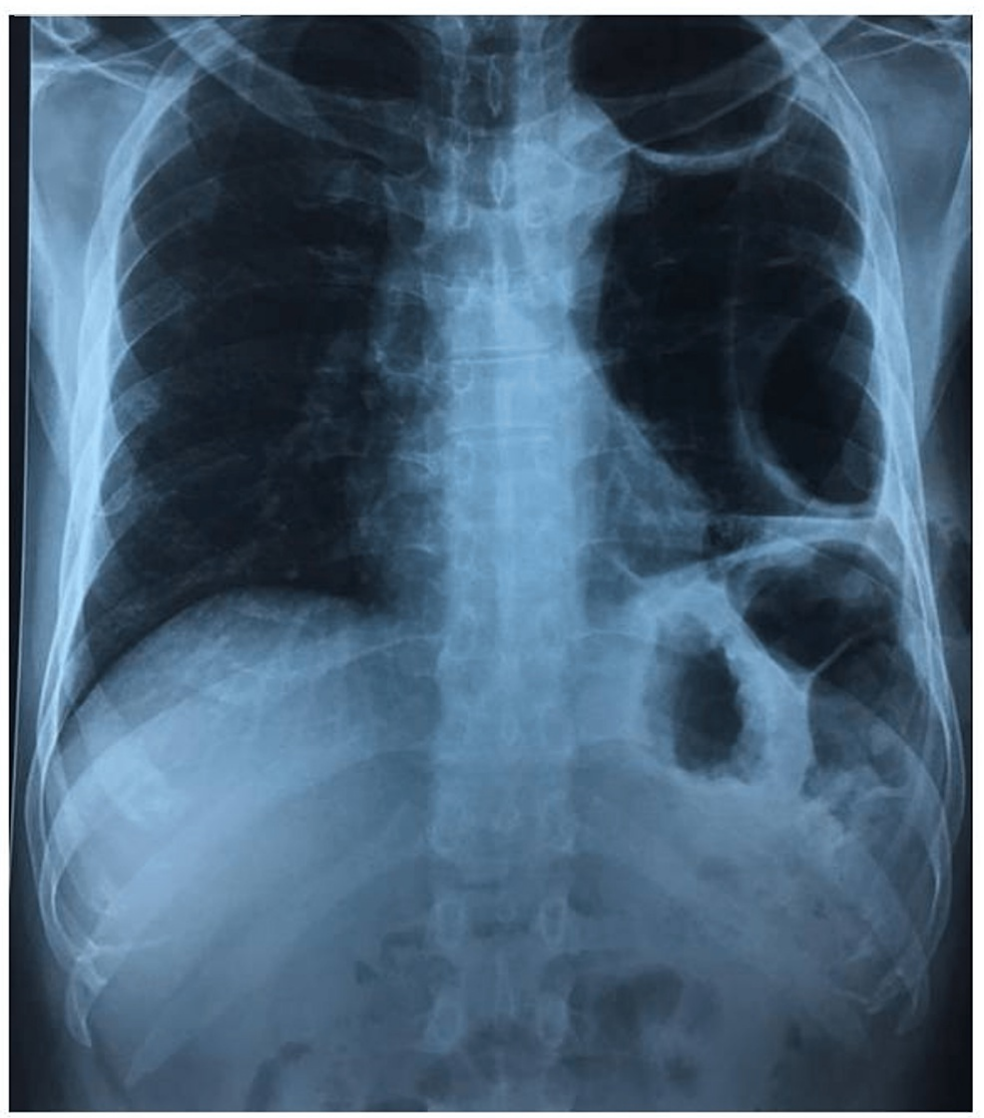
Chest radiograph showing a left-sided hydropneumothorax with the collapse of the left lung and mediastinal shift to the right suggestive of left pleural empyema

A chest radiograph revealed a left-sided hydropneumothorax with a collapse of the left lung and a mediastinal shift to the right suggestive of left pleural empyema (Figure 1). The aspirated thick pleural pus obtained on thoracocentesis was submitted for microbiological investigations, including bacterial culture. The patient was empirically started on parenteral antibiotics (piperacillin-tazobactam 4.5 g twice daily, clindamycin 300 mg twice daily, and metronidazole 500 mg thrice daily) along with intercostal tube drainage and supportive therapy for correction of anemia pending culture results. Further cycles of chemotherapy were deferred till correction of the pleural lesion.

**Figure 2:**
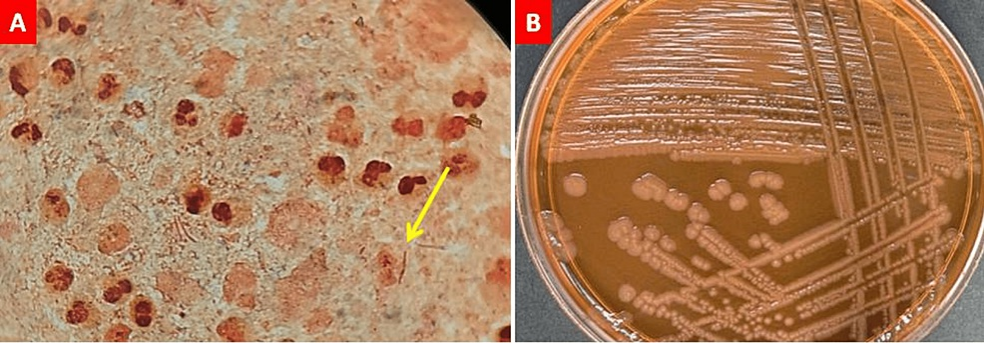
Pleural pus aspirate showing (A) numerous pus cells (1000×) and many gram-negative rods, and (B) growth of Proteus mirabilis on MacConkey agar plate

The gram-stained smear showed numerous polymorphonuclear leukocytes (>100/low power field) with many gram-negative rods (Figure 2, Panel A). The culture yielded pure growth of translucent non-lactose fermenting colonies on the MacConkey agar plate (Figure 2, Panel B) and swarming growth on the blood agar plate identified as *P. mirabilis* by an automated platform (VITEK® 2 GN, bioMérieux, Marcy l’Etoile, France). The isolate was susceptible to third- and fourth-generation cephalosporins, piperacillin-tazobactam, and carbapenems but resistant to fluoroquinolones (ciprofloxacin and levofloxacin), aminoglycosides (amikacin and netilmicin), and trimethoprim-sulfamethoxazole. Other routine microbiological investigations including blood and urine cultures as well as cartridge-based nucleic acid amplification tests of the pus sample for tuberculosis were non-contributory. Antibiotic therapy with intravenous piperacillin-tazobactam was continued, while clindamycin and metronidazole were withdrawn. Tube drainage and other supportive treatment were continued. The patient improved gradually, the fever and pleuritic pain subsided, and the effusion resolved progressively. A repeat culture of the resolving pus aspirate from the drain tube at two weeks of therapy showed no bacterial growth. Antibiotics were changed to oral cefixime (200 mg every 12 hours) for one week. He was discharged after three weeks of hospitalization with advice for regular follow-up at the hospital for further planned management for chemotherapy.

## Discussion

Pleural infection, characterized by pus or bacteria in the pleural space, continues to cause significant morbidity and mortality worldwide, despite years of learned experience, advances in modern healthcare, and availability of newer antibiotics [4,5]. An additional burden is the associated cost of hospitalization. As per an estimate, about 65,000 patients with pleural infections require hospitalization in the United Kingdom and the United States, which accounts for a phenomenal cost of US$ 500 million per year [4]. A recent report suggests an increasing incidence of empyema cases [5].

An insight into the bacteriology of pleural empyema revealed *Streptococcus milleri* as the most common isolate accounting for 30%-50% of adult cases of community-acquired empyema followed by *Streptococcus pneumoniae* and anaerobes, while *Staphylococcus aureus* was the most common isolate for hospital-acquired cases [4,5]. Other reports suggested *Klebsiella pneumoniae* as the commonest organism of empyema in both community- and hospital-acquired cases [4,5]. Pleural space infections due to *Proteus *species are infrequent, with the majority of cases occurring in immunocompromised patients such as malnutrition, malignancy, and chronic liver and kidney diseases.

We searched the PubMed database in English literature from 1960 for pleural space infections caused by *Proteus *species in adults with the keywords “Pleural empyema and Proteus,” “Pleural effusion and Proteus,” “Pleural empyema and gram-negative bacilli,” “Pleural effusion and gram-negative bacilli,” “parapneumonic pleural effusion and gram-negative bacilli,” “pleural space infection and gram-negative bacilli,” and “parapneumonic pleural effusion and infectious etiology” and extracted data pertaining only to the adult population, where possible. The search uncovered 49 previously documented cases of pleural space infection (effusion and/or empyema) caused by *Proteus *species in adults (Table 2) [6-20].

**Table 2:** Pleural space infection caused by Proteus species in adults USA: United States of America; tPA: Tissue plasminogen activator; DNAse: Deoxyribonuclease; VATS: Video-assisted thoracoscopic surgery; NA: Data not available.

Year, Country, [Reference]	No. of cases	Age (in years)	Sex	Associated condition/s	Hemithorax involved	Proteus species	Other organisms isolated	Antibiotic therapy	Other treatment	Outcome
Adults										
1974, USA [6]	2	Adults	NA	NA	NA	P. mirabilis	NA	NA	NA	NA
1983, USA [7]	1	70	F	Advanced breast cancer with metastatic disease to the skin, bone marrow, and pleura	Left	P. mirabilis	*Staphylococcus aureus*, *Peptococcus asaccharolyticus*, and *Bacteroides melaninogenicus*	Cephalothin and gentamicin	Chest tube drainage	Died
1983, USA [7]	1	63	M	Laryngeal carcinoma, cholecystectomy for gallbladder stones	Bilateral, more on right	P. mirabilis	*Fusobacterium nucleatum*, *Propionibacterium acnes*, and alpha-hemolytic *Streptococcus*	Ampicillin and other antibiotics	Chest tube drainage	Recovered
1983, USA [7]	1	63	F	Myocardial infarction, left ventricular aneurysm, and refractory congestive heart failure	Left	P. mirabilis	Group D *Streptococcus *(nonenterococcus), *Bacteroides distatonis*, anaerobic Gram-positive coccus, and *Bacteroides bivius*	Cefazolin	Chest tube drainage	Recovered
1990, USA [8]	1	55	M	None	Right	P. vulgaris	Enterococcus	Gentamicin, penicillin, and cefoxitin	Chest tube drainage	Recovered
1995, USA [9]	3	33-88	NA	NA	NA	NA	NA	NA	NA	NA
2006, Taiwan [10]	3	22-92	NA	Intensive care unit patients	NA	P. mirabilis	NA	NA	NA	NA
2007, Taiwan [11]	12	NA	NA	NA	NA	NA	NA	NA	NA	NA
2007, Taiwan [12]	3	18-44	NA	NA	NA	*Proteus *spp.	NA	NA	NA	NA
2007, Taiwan [13]	3	Adults	NA	Chronic liver disease and end-stage renal disease	NA	P. mirabilis	NA	NA	NA	NA
2007, India [14]	2	Adults	NA	NA	NA	P. mirabilis	NA	NA	NA	NA
2008, Taiwan [15]	6	Adults	NA	Medical ward and medical intensive care unit patients	NA	P. mirabilis	NA	NA	NA	NA
2009, Taiwan [16]	5	22-87	NA	NA	NA	P. mirabilis	NA	NA	NA	NA
2010, India [17]	2	>15	NA	NA	NA	NA	NA	NA	NA	Recovered
2018, Belgium [18]	1	74	M	Boerhaave’s syndrome	Left	P. mirabilis	Saccharomyces cerevisiae	Meropenem, vancomycin, and liposomal amphotericin B	Chest tube drainage	Recovered
2020, USA [19]	1	40	F	Asthma, type 2 diabetes, sickle cell trait, and infected renal cyst	Left	P. mirabilis	NA	Ceftriaxone and metronidazole	Chest tube, intrapleural fibrinolytics with tPA and DNAse, and VATS decortication of the left visceral pleura	Recovered
2021, Greece [20]	2	>18	NA	NA	NA	P. mirabilis	NA	NA	NA	NA
Current case										
2021, India	1	44	M	Adenocarcinoma tongue	Left	P. mirabilis	None	Piperacillin-tazobactam	Chest tube drainage	Recovered

Of 29 cases documenting the specific species, *P. mirabilis* was found in 28 (96.5%) cases and *P. vulgaris* (3.4%) was found in one. Common comorbidities included intensive care unit admission in nine cases, chronic liver and renal diseases in three cases, and malignancy in two patients [7,10,13,15]. One patient had multiple comorbidities of asthma, type 2 diabetes, sickle cell trait, and an infected renal cyst [19]. Others had severe cardiac disease [7] and Boerhaave’s syndrome [18]. Out of eight cases with documented information on the outcome, the majority (seven) had recovered (87.5%), while one (12.5%) patient died. In the present case, the patient was an adult, with adenocarcinoma of the tongue as an underlying comorbidity. The isolate was susceptible to the majority of antimicrobials, and the patient had a successful outcome. To our knowledge, this is probably the first case of *Proteus *empyema associated with oral cancer. The source of infection can only be speculated, with possible translocation of the organism from the gastrointestinal tract or inoculation through a breach in the skin during the previous cycle of chemotherapy, giving rise to transient bacteremia and seeding into the pleural cavity.

## Conclusions

Though uncommon, the possibility of *Proteus *species should be kept in mind as a causative agent of thoracic empyema, especially in immunocompromised patients with cancer, diabetes, and renal diseases. The so-called common microorganisms of empyema appear to have altered over time, possibly influenced by anticancer therapy and underlying host immune status. Reliable identification and speciation of culture-positive isolates are crucial for administering appropriate antibiotics and assuring a good outcome.

## References

[1] Senior BW (2005). Proteus, morganella, and providencia. Topley and Wilson's Microbiology and Microbial Infections: Parasitology, 10th Edition.

[2] Drzewiecka D (2016). Significance and roles of proteus spp. bacteria in natural environments. Microb Ecol.

[3] Palusiak A (2022). Proteus mirabilis and Klebsiella pneumoniae as pathogens capable of causing co-infections and exhibiting similarities in their virulence factors. Front Cell Infect Microbiol.

[4] Lee P (2019). Empyema: a debilitating condition that warrants further research. Respirology.

[5] Burgos J, Falcó V, Pahissa A (2013). The increasing incidence of empyema. Curr Opin Pulm Med.

[6] Bartlett JG, Gorbach SL, Thadepalli H, Finegold SM (1974). Bacteriology of empyema. Lancet.

[7] Pine JR, Hollman JL (1983). Elevated pleural fluid pH in Proteus mirabilis empyema. Chest.

[8] Isenstein D, Honig E (1990). Proteus vulgaris empyema and increased pleural fluid pH. Chest.

[9] Civen R, Jousimies-Somer H, Marina M, Borenstein L, Shah H, Finegold SM (1995). A retrospective review of cases of anaerobic empyema and update of bacteriology. Clin Infect Dis.

[10] Tu CY, Hsu WH, Hsia TC, Chen HJ, Chiu KL, Hang LW, Shih CM (2006). The changing pathogens of complicated parapneumonic effusions or empyemas in a medical intensive care unit. Intensive Care Med.

[11] Lin YC, Tu CY, Chen W, Tsai YL, Chen HJ, Hsu WH, Shih CM (2007). An urgent problem of aerobic gram-negative pathogen infection in complicated parapneumonic effusions or empyemas. Intern Med.

[12] Liang SJ, Chen W, Lin YC, Tu CY, Chen HJ, Tsai YL, Shih CM (2007). Community-acquired thoracic empyema in young adults. South Med J.

[13] Chen CH, Hsu WH, Chen HJ, Chen W, Shih CM, Hsia TC, Tu CY (2007). Different bacteriology and prognosis of thoracic empyemas between patients with chronic and end-stage renal disease. Chest.

[14] Malhotra P, Aggarwal AN, Agarwal R, Ray P, Gupta D, Jindal SK (2007). Clinical characteristics and outcomes of empyema thoracis in 117 patients: a comparative analysis of tuberculous vs. non-tuberculous aetiologies. Respir Med.

[15] Lin YC, Chen HJ, Liu YH, Shih CM, Hsu WH, Tu CY (2008). A 30-month experience of thoracic empyema in a tertiary hospital: emphasis on differing bacteriology and outcome between the medical intensive care unit (MICU) and medical ward. South Med J.

[16] Chen W, Lin YC, Liang SJ (2009). Hospital-acquired thoracic empyema in adults: a 5-year study. South Med J.

[17] Kundu S, Mitra S, Mukherjee S, Das S (2010). Adult thoracic empyema: a comparative analysis of tuberculous and nontuberculous etiology in 75 patients. Lung India.

[18] Teblick A, Jansens H, Dams K, Somville FJ, Jorens PG (2018). Boerhaave's syndrome complicated by a Saccharomyces cerevisiae pleural empyema. Case report and review of the literature. Acta Clin Belg.

[19] Earasi K, Welch C, Zelickson A, Westover C, Ramani C, Sumner C, Davis EM (2020). Proteus empyema as a rare complication from an infected renal cyst, a case report. BMC Pulm Med.

[20] Iliopoulou M, Skouras V, Psaroudaki Z (2021). Bacteriology, antibiotic resistance and risk stratification of patients with culture-positive, community-acquired pleural infection. J Thorac Dis.

